# Instruments for Assessing Risk of Bias and Other Methodological Criteria: Krauth et al. Respond

**DOI:** 10.1289/ehp.1307727R

**Published:** 2014-03-01

**Authors:** David Krauth, Tracey J. Woodruff, Lisa Bero

**Affiliations:** 1 Department of Clinical Pharmacy, and; 2 Department of Obstetrics, Gynecology, and Reproductive Sciences, University of California, San Francisco, San Francisco, California, USA; 3 Program on Reproductive Health and the Environment, Oakland, California, USA; 4 Institute for Health Policy Studies, University of California, San Francisco, San Francisco, California, USA

Beck et al. criticize our systematic review (Krauth et al. 2013) because we included instruments derived from preclinical animal research. Assessment instruments developed for preclinical animal models have criteria that are relevant to hazard and risk assessment because risk of bias in animal studies is not dependent on the data stream or the question being asked, but on the design of the study. Many instruments that have been developed (including those for evaluating animal toxicology studies) have criteria that have not been shown to bias research outcomes (see Supplemental Material, Table S1, of Krauth et al. 2013).

Furthermore, Table 1 of our paper (Krauth et al. 2013) lists the criteria found in most instruments we identified. In the “Discussion,” we described the empirical evidence supporting the use of some of these criteria and cited the relevant references with the empirical data. By empirical evidence, we mean that a criterion (e.g., randomization) has been shown to be associated with overestimation or underestimation of effect (this could be an efficacy or harm outcome).

Beck et al. note several publications in environmental chemical health hazard assessment [Ågerstrand et al. 2011; Food and Drug Administration (FDA) 2003; Hulzebos et al. 2010; Organisation for Economic Co-operation and Development (OECD) 1998; Schneider et al. 2009; U.S. Environmental Protection Agency (EPA) 1999a, 1999b, 2013]. All of these publications, except OECD (1998), were identified in our search; however, they did not meet the *a priori* inclusion criteria for our systematic review. As noted in our “Methods” (Krauth et al. 2013), we included the earliest publication of an instrument when it was used in subsequent reports. The article by Ågerstrand et al. (2011) was based on four earlier published papers (i.e., Durda and Preziosi 2000; Hobbs et al. 2005; Klimisch et al. 1997; Schneider et al. 2009). We cited three of these in our review, but excluded Schneider et al. (2009) because it appeared to be a description of software that could be used to operationalize the Klimisch criteria. After reviewing the criteria described by Schneider et al. (2009) in their supplemental file, we found no unique additional criteria that were not already included in our Table 1 and Supplemental Material, Table S1. The reports from the U.S. EPA (1999a, 1999b) and FDA (2003) were neither indexed in Medline nor found in screening of bibliographies. In addition, U.S. EPA (2013) was published after we ended our study. Because we did not find the OECD document (OECD 1998), we cannot conclude whether or not it should have been included in our study.

The comment by Beck et al. that the National Toxicology Program is relying on criteria that have not been “transparently empirically tested” is not correct. In our paper (Krauth et al. 2013), we recommended the use of empirically tested criteria and we pointed out criteria that have been shown to be a risk of bias.

We caution against gathering judgments on how to assess study quality and propose that evidence should guide such evaluations. We propose an empirically based approach—as opposed to consensus-based opinion of experts—as this would provide a more unbiased evaluation of the data.
